# New Parameterized Inequalities for *η*-Quasiconvex Functions via (*p*, *q*)-Calculus

**DOI:** 10.3390/e23111523

**Published:** 2021-11-16

**Authors:** Humaira Kalsoom, Miguel Vivas-Cortez, Muhammad Idrees, Praveen Agarwal

**Affiliations:** 1 Department of Mathematics, Zhejiang Normal University, Jinhua 321004, China; humaira87@zju.edu.cn or; 2 Escuela de Ciencias Físicas y Matemáticas, Facultad de Ciencias Naturales y Exactas, Pontificia Universidad Católica del Ecuador, Quito 17-01-2184, Ecuador; 3 Zhejiang Province Key Laboratory of Quantum Technology and Device, Department of Physics, Hangzhou 310027, China; Idrees@zju.edu.cn; 4 Department of Mathematics, Anand International College of Engineering, Jaipur 302001, India; goyal.praveen2011@gmail.com

**Keywords:** quantum calculus, post quantum calculus, parameterized (*p*, *q*)-estimates for midpoint and trapezoidal type inequalities, *η*-quasiconvexity

## Abstract

In this work, first, we consider novel parameterized identities for the left and right part of the (p,q)-analogue of Hermite–Hadamard inequality. Second, using these new parameterized identities, we give new parameterized (p,q)-trapezoid and parameterized (p,q)-midpoint type integral inequalities via η-quasiconvex function. By changing values of parameter μ∈[0,1], some new special cases from the main results are obtained and some known results are recaptured as well. Finally, at the end, an application to special means is given as well. This new research has the potential to establish new boundaries in comparative literature and some well-known implications. From an application perspective, the proposed research on the η-quasiconvex function has interesting results that illustrate the applicability and superiority of the results obtained.

## 1. Introduction

Quantum calculus, usually referred as *q*-calculus, is a numerical technique that examines calculus without limits. The genius who created the analytical *q*-calculus in the eighteenth century was the great mathematician L. Euler, who integrated the parameter *q* into Newton’s work of infinite series. Jackson [1] is credited with being the first to define and study the *q*-integral in a systematic manner, back in the early twentieth century. The fundamental goal of q-calculus is to find the q-analogues of mathematical objects recovered by taking $q\to 1^{-}$. However, in order to keep up with the trends, it has experienced remarkable expansion during the last few decades. The q-calculus has gained popularity in recent years due to its versatility in subjects like mathematics and physics. In 2002, V. Kac and P. Cheung [2] published a book in which they explained the main fundamental concept of the *q*-calculus in a concise manner. In 2004, Gauchman [3] introduced the concept of quantum-integral inequalities in the theory of quantum calculus as well as their applications and results in these fields of study. In 2012, Ernst [4] proposed a comprehensive treatment of *q*-calculus which is great achievement in the field of mathematical inequalities.

In 2013, J. Tariboon and S. K. Ntouyas [5] published a study describing the essential features of innovative quantum-derivatives and quantum-integrals are proved over $[\epsilon_{1},\epsilon_{2}]\subset R$. First- and second-order impulsive *q*-differential equations were examined, as well as initial value problems in these processes. Some of the inequalities to which these definitions apply include Hermite–Hadamard inequalities, Fejér type inequalities, Simpson type inequalities, Newton type inequalities, Ostrowski type inequalities, among others, for more details see in [6,7,8,9,10,11,12,13,14,15,16] and the references cited therein.

The post-quantum calculus, alternatively referred to as the (p,q)-calculus, is a generalization of the *q*-calculus on the interval (0,∞). The (p,q)-calculus is made up of two-parameter quantum calculus (*p* and *q* numbers) that are completely independent of one another. R. Chakrabarti and R. A. Jagannathan [17] were the first to propose the (p,q)-calculus, which was published in 1991. Later, M. Tunc and E. Göv [18] refined the new (p,q)-derivative and (p,q)-integral of a arbitrary function on a finite interval in 2016. There have been numerous other (p,q)-analogs of classical inequalities uncovered throughout the years. For the case of p=1, we get the *q*-calculus formula, and for the case of $q\to 1^{-}$, we get the classical formula. Kalsoom et al. [19] and Kunt et al. [20] showed that the left side of the (p,q)-midpoint inequality can be proved using (p,q)-differentiable convex and quasi-convex functions, and then developed some novel (p,q)-Hermite–Hadamard inequality. Utilizing an unique integral identity with (p,q)-differentiable functions, Latif et al. [21] discovered some new forms of post-quantum trapezoid type inequalities that were previously unknown. Using (α,m)-convex mappings, Humaira et al. [22] established the idea of (p,q)-estimates for distinct types of integral inequalities, for more details see in [23,24,25,26,27,28] and the references cited therein.

As mathematical inequalities have several applications in both mathematics and physics, they are crucial to the study of mathematics as well as other branches of mathematics.

Let H:I⊆R→R be a convex function, if

$$H\left(\pi \epsilon_{1}+\left(1-\pi \right)\epsilon_{2}\right)\leq \pi H\left(\epsilon_{1}\right)+\left(1-\pi \right)H\left(\epsilon_{2}\right)$$

for all $\epsilon_{1},\epsilon_{2}\in I$ and π∈[0,1].

Convexity in relation to integral inequalities is an intriguing area of study. Many inequalities arise as a direct result of the use of convex functions. One of the most important results in convex analysis is the Hermite–Hadamard type inequality, which offers a necessary and sufficient condition for a function to be convex. This classic Hermite and Hadamard result is as follows.

If H:I⊆R→R be a convex function on the interval I with $\epsilon_{1}<\epsilon_{2},$ then

(1) $$H\left(\frac{\epsilon_{1}+\epsilon_{2}}{2}\right)\leq \frac{1}{\epsilon_{2}-\epsilon_{1}}\int_{\epsilon_{1}}^{\epsilon_{2}}H\left(\pi \right)d\pi \leq \frac{H\left(\epsilon_{1}\right)+H\left(\epsilon_{2}\right)}{2}.$$

Equation (1) was first introduced by C. Hermite [29] in 1893, and it was further researched by J. Hadamard [30] the following year. Both inequalities hold in the inverted direction if H is concave, which implies that it is. A large number of mathematicians have given close attention to the Hermite–Hadamard inequality because of its high quality and consistency in the field of mathematical inequality. There have been important advancements, revisions, and ramifications in the Hermite–Hadamard uniqueness property as well as broader convex function definitions. Dragomir et al. [31] proposed two inequalities for differentiable mappings and applications to special means of real numbers and to trapezoidal formula. Sarikaya et al. [32] established Hermite–Hadamard type inequality for convex function, for more details see in [33,34,35,36,37,38,39,40] and the references cited therein.

Gordji et al. [41,42] have presented a new class of functions known as an η-quasiconvex functions.

**Definition** **1.** *A function H:I⊂R→R is considered an η-quasiconvex functions with respect to η:R×R→R, if*

$$H\left(\pi \epsilon_{1}+\left(1-\pi \right)\epsilon_{2}\right)\leq \max\left\{H\left(\epsilon_{2}\right),H\left(\epsilon_{2}\right)+\eta \left(H\left(\epsilon_{1}\right),H\left(\epsilon_{2}\right)\right)\right\}$$

*holds for all $\epsilon_{1},\epsilon_{2}\in I$ and $\pi \in \left[0,1\right]$.*

Inspired by the ongoing studies, we give the generalizations of the results proved in [20,21] and we prove parameterized (p,q)-estimates of midpoint and trapezoidal type inequalities for η-quasiconvex functions using the concepts of ${}_{\epsilon_{1}}D_{p,q}$-difference operator and ${\left(p,q\right)}_{\epsilon_{1}}$-integral.

This paper is organized as follows. In Section 2, we provide a brief introduction of the principles of *q*-calculus and (p,q)-calculus, as well as other related studies in this area. In Section 3, we give some novel parameterized (p,q) estimates of trapezoidal and midpoint type inequalities for eta-quasiconvex functions, as well as a comparison of the results presented here with analogous results in the literature. We also present several applications to special means in Section 4 to demonstrate our new methodology. The conclusion is offered in Section 5 at the end of this work.

## 2. Preliminaries of q, (p,q)-Calculus and Some Inequalities

This section of the paper will discuss in detail the principles of *q* and (p,q) calculus, as well as several significant *q* and (p,q) midpoint and *q* and (p,q) trapezoidal integral inequalities. Throughout this work, we shall refer to the constants 0<q<p≤1.

The ${\left[n\right]}_{q}$ is said to be *q*-integers and is expressed as

(2) $${\left[n\right]}_{q}=1+q+q^{2}+\cdots +q^{n-1}=\frac{1-q^{n}}{1-q}$$

for n∈N and ${\left[n\right]}_{q}=n$ for n=1.

The *q*-factorial and for 0≤k≤n, the *q*-binomial are defined as follows:

$$\begin{array}{cc} {\left[n\right]}_{q}! & =\prod_{m=1}^{n}{\left[m\right]}_{q},n\geq 1,\;{\left[0\right]}_{q}!=1,\\ {\left[\begin{array}{c} n\\ k \end{array}\right]}_{q}! & =\frac{{\left[n\right]}_{q}!}{{\left[n-k\right]}_{q}!{\left[k\right]}_{q}!}. \end{array}$$

During a period in the early twentieth century, Jackson made substantial changes to the classical notion of a derivative of a function, enabling a more straightforward study of fundamental calculus and number theory in this examination. The development of *q*-analogues of some of the most significant discoveries made in these fields is attributed to Jackson, who is also credited with the publication of certain seminal papers in the field, such as [1]

(3) $$D_{q}H\left(\epsilon \right)=\frac{H\left(\epsilon \right)-H\left(q\epsilon \right)}{\left(1-q\right)\epsilon },\;\epsilon \neq 0.$$

The classic Jackson integral of a real function H is defined by the following series expansion:

(4) $$\int_{0}^{\epsilon_{2}}H\left(\epsilon \right)d_{q}\epsilon =\left(1-q\right)\epsilon_{2}\sum_{n=0}^{\infty }q^{n}H\left(\epsilon_{2}q^{n}\right)$$

provided the sum converge absolutely.

The *q*-Jackson integral in a generic interval $\left[\epsilon_{1},\epsilon_{2}\right]$ is defined as follows:

$$\int_{\epsilon_{1}}^{\epsilon_{2}}H\left(\epsilon \right)d_{q}\epsilon =\int_{0}^{\epsilon_{2}}H\left(\epsilon \right)d_{q}\epsilon -\int_{0}^{\epsilon_{1}}H\left(\epsilon \right)d_{q}\epsilon .$$

The *q*-analogues of these number theory, deduction, and ordinary integration conclusions are polynomial expressions in a real variable *q* that are reduced to the classical ideas when *q* approaches to 1.

**Definition** **2.** [5] *Suppose that a function $H:\left[\epsilon_{1},\epsilon_{2}\right]\to R$ is continuous. Then, the $q_{\epsilon_{1}}$-derivative of H at ϵ is defined as follows:*

(5) $${}_{\epsilon_{1}}D_{q}H\left(\epsilon \right)=\frac{H\left(\epsilon \right)-H\left(q\epsilon +\left(1-q\right)\epsilon_{1}\right)}{\left(1-q\right)\left(\epsilon -\epsilon_{1}\right)},\;\epsilon \neq \epsilon_{1}.$$

*As H is a continuous function from $\left[\epsilon_{1},\epsilon_{2}\right]$ to R, so for $\epsilon =\epsilon_{1}$, we define ${}_{\epsilon_{1}}D_{q}H\left(\epsilon_{1}\right)=\lim_{\epsilon \to \epsilon_{1}}D_{q}H\left(\epsilon \right)$, if ${}_{\epsilon_{1}}D_{q}H\left(\epsilon \right)$ exists for all $\epsilon \in \left[\epsilon_{1},\epsilon_{2}\right]$, then the function H is called $q_{\epsilon_{1}}$-differentiable on $\left[\epsilon_{1},\epsilon_{2}\right].$*

**Remark** **1.** *It is important to remember that, if $\epsilon_{1}=0$ in* (5)*, we get the equivalent q-derivative defined in* (3).

**Lemma** **1.** [5] *Let α∈R, we have*

$${}_{\epsilon_{1}}D_{q}{\left(x-\epsilon_{1}\right)}^{\alpha }=\left(\frac{1-q^{\alpha }}{1-q}\right){\left(x-\epsilon_{1}\right)}^{\alpha -1}.$$

**Definition** **3.** [5] *Suppose that a function $H:\left[\epsilon_{1},\epsilon_{2}\right]\to R$ is continuous, then the $q_{\epsilon_{1}}$-definite integral of H at $\left[\epsilon_{1},\epsilon_{2}\right]$ is defined as follows:*

(6) $$\int_{\epsilon_{1}}^{x}H{\left(\epsilon \right)}_{\epsilon_{1}}d_{q}\epsilon =\left(1-q\right)\left(x-\epsilon_{1}\right)\sum_{n=0}^{\infty }q^{n}H\left(q^{n}x+\left(1-q^{n}\right)\epsilon_{1}\right),\;x\in \left[\epsilon_{1},\epsilon_{2}\right].$$

The following results hold about definite $q_{\epsilon_{1}}$-integrals.

**Theorem** **1.** [5] *Let H:I→R be a continuous function. Then,*

*1.* 
${}_{\epsilon_{1}}D_{q}\int_{\epsilon_{1}}^{x}H\left(\pi \right){\;}_{\epsilon_{1}}d_{q}\pi =H\left(x\right);$

*2.*  *$\int_{\epsilon_{3}}^{x}$${}_{\epsilon_{1}}D_{q}H\left(\pi \right){\;}_{\epsilon_{1}}d_{q}\pi =H\left(x\right)-H\left(\epsilon_{3}\right)$, $\epsilon_{3}\in \left(\epsilon_{1},x\right).$*

**Theorem** **2.** [5] *Suppose that H,G:I→R are continuous functions, α∈R. Then, for x∈I,*

*1.* 
$\int_{\epsilon_{1}}^{x}\left[H\left(\pi \right)+G\left(\pi \right)\right]{\;}_{\epsilon_{1}}d_{q}\pi =\int_{\epsilon_{1}}^{x}H{\left(t\right)}_{\epsilon_{1}}d_{q}\pi +\int_{\epsilon_{1}}^{x}G\left(\pi \right){\;}_{\epsilon_{1}}d_{q}\pi ;$

*2.* 
$\int_{\epsilon_{1}}^{x}\alpha H\left(\pi \right){\;}_{\epsilon_{1}}d_{q}\pi =\alpha \int_{\epsilon_{1}}^{x}H\left(\pi \right){\;}_{\epsilon_{1}}d_{q}\pi ;$

*3.*  *$\int_{\epsilon_{3}}^{x}H\left(\pi \right){\;}_{\epsilon_{1}}D_{q}G\left(\pi \right){\;}_{\epsilon_{1}}d_{q}\pi ={\left.H\left(\pi \right)G\left(\pi \right)\right|}_{\epsilon_{3}}^{x}-\int_{\epsilon_{3}}^{x}G{\left(q\pi +\left(1-q\right)\epsilon_{1}\right)}_{\epsilon_{1}}D_{q}H\left(\pi \right){\;}_{\epsilon_{1}}d_{q}\pi$, $\epsilon_{3}\in \left(\epsilon_{1},x\right)$.*

The $q_{\epsilon_{1}}$-Hermite–Hadamard inequalities for convexity were discovered by Alp and colleagues in [14], and they are defined as follows:

**Theorem** **3.** *Let $H:\left[\epsilon_{1},\epsilon_{2}\right]\to R$ be a convex differentiable function on $\left(\epsilon_{1},\epsilon_{2}\right)$. Then, the following are the $q_{\epsilon_{1}}$-Hermite–Hadamard integral inequalities:*

(7) $$H\left(\frac{q\epsilon_{1}+\epsilon_{2}}{{\left[2\right]}_{q}}\right)\leq \frac{1}{\epsilon_{2}-\epsilon_{1}}\int_{\epsilon_{1}}^{\epsilon_{2}}H{\left(\epsilon \right)}_{\epsilon_{1}}d_{q}\epsilon \leq \frac{qH\left(\epsilon_{1}\right)+H\left(\epsilon_{2}\right)}{{\left[2\right]}_{q}}.$$

**Lemma** **2.** [5] *For $\alpha \in R\backslash \left\{-1\right\}$, the following formula holds:*

$$\int_{\epsilon_{1}}^{x}{\left(\pi -\epsilon_{1}\right)}^{\alpha }{\;}_{\epsilon_{1}}d_{q}\pi =\left(\frac{1-q}{1-q^{\alpha +1}}\right){\left(x-\epsilon_{1}\right)}^{\alpha +1}.$$

**Remark** **2.** *If we take $\epsilon_{1}=0$ in* (6)*, we obtain the classicalq-integral defined in* (4).

## 3. (p,q)-Derivatives and Integrals

The post-quantum calculus, alternatively referred to as the (p,q)-calculus is the generalization of *q*-calculus. In this section, we review some fundamental notions and notations of $\left(p,q\right)$-calculus.

The ${\left[n\right]}_{p,q}$ is said to be (p,q)-integers and is expressed as

$${\left[n\right]}_{p,q}=\frac{p^{n}-q^{n}}{p-q}.$$

The (p,q)-factorial and for 0≤k≤n, the (p,q)-binomial are defined as follows:

$$\begin{array}{cc} {\left[n\right]}_{p,q}! & =\prod_{m=1}^{n}{\left[m\right]}_{p,q},n\geq 1,\;{\left[0\right]}_{p,q}!=1,\\ {\left[\begin{array}{c} n\\ k \end{array}\right]}_{p,q}! & =\frac{{\left[n\right]}_{p,q}!}{{\left[n-k\right]}_{p,q}!{\left[k\right]}_{p,q}!}. \end{array}$$

**Definition** **4.** [24] *Suppose that a function $H:\left[\epsilon_{1},\epsilon_{2}\right]\to R$, then $\left(p,q\right)$-derivative is defined as*

(8) $$D_{p,q}H\left(\epsilon \right)=\frac{H\left(p\epsilon \right)-H\left(q\epsilon \right)}{\left(p-q\right)\epsilon },\;\epsilon \neq 0.$$

**Definition** **5.** [18] *If $H:[\epsilon_{1},\epsilon_{2}]\to R$ is a continuous function then the (p,q)-derivative of H at $\epsilon \in [\epsilon_{1},\epsilon_{2}]$ is defined as*

(9) $${}_{\epsilon_{1}}D_{p,q}H\left(\epsilon \right)=\frac{H\left(p\epsilon +\left(1-p\right)\epsilon_{1}\right)-H\left(q\epsilon +\left(1-q\right)\epsilon_{1}\right)}{\left(p-q\right)\left(\epsilon -\epsilon_{1}\right)},\;\epsilon \neq \epsilon_{1}.$$

*As H is a continuous function from $\left[\epsilon_{1},\epsilon_{2}\right]$ to R, so for $\epsilon =\epsilon_{1}$, we define ${}_{\epsilon_{1}}D_{p,q}H\left(\epsilon_{1}\right)=\lim_{\epsilon \to \epsilon_{1}}D_{p,q}H\left(\epsilon \right)$, if ${}_{\epsilon_{1}}D_{p,q}H\left(\epsilon \right)$ exists for all $\epsilon \in \left[\epsilon_{1},\epsilon_{2}\right]$, then the function H is called ${\left(p,q\right)}_{\epsilon_{1}}$-differentiable on $\left[\epsilon_{1},\epsilon_{2}\right].$*

The following result is very important to evaluate ${\left(p,q\right)}^{\epsilon_{2}}$-derivative at $\epsilon \in \left[\epsilon_{1},\epsilon_{2}\right].$

**Remark** **3.** *It is important to remember that, if $\epsilon_{1}=0$ in* (9)*, we get the equivalent* (p,q)*-derivative defined in* (8).

**Definition** **6.** [18] *Suppose that a function $H:\left[\epsilon_{1},\epsilon_{2}\right]\to R$ is continuous, then the ${\left(p,q\right)}_{\epsilon_{1}}$-definite integral of H at $\left[\epsilon_{1},\epsilon_{2}\right]$ is defined as follows: *

(10) $$\int_{\epsilon_{1}}^{x}H\left(\epsilon \right){\;}_{\epsilon_{1}}d_{p,q}\epsilon =\left(p-q\right)\left(x-\epsilon_{1}\right)\sum_{n=0}^{\infty }\frac{q^{n}}{p^{n+1}}H\left(\frac{q^{n}}{p^{n+1}}x+\left(1-\frac{q^{n}}{p^{n+1}}\right)\epsilon_{1}\right),\;x\in \left[\epsilon_{1},\epsilon_{2}\right].$$

The following ${\left(p,q\right)}_{\epsilon_{1}}$ result was proven by Kunt and colleagues in [20]. Inequalities of the kind Hermite–Hadamard for convex functions obtained by using the ${\left(p,q\right)}_{\epsilon_{1}}$-integral:

**Theorem** **4.** *For a convex mapping $H:\left[\epsilon_{1},\epsilon_{2}\right]\to R$ which is differentiable on $\left(\epsilon_{1},\epsilon_{2}\right),$ then the essential inequalities are given below:*

(11) $$H\left(\frac{q\epsilon_{1}+p\epsilon_{2}}{{\left[2\right]}_{p,q}}\right)\leq \frac{1}{p\left(\epsilon_{2}-\epsilon_{1}\right)}\int_{\epsilon_{1}}^{p\epsilon_{2}+\left(1-p\right)\epsilon_{1}}H\left(x\right){\;}_{\epsilon_{1}}d_{p,q}x\leq \frac{qH\left(\epsilon_{1}\right)+pH\left(\epsilon_{2}\right)}{{\left[2\right]}_{p,q}}.$$

**Remark** **4.** *If we take $\epsilon_{1}=0$ and $x=\epsilon_{2}=1$ in* (10)*, then we have*

$$\int_{0}^{1}H\left(\epsilon \right)\;d_{p,q}\epsilon =\left(p-q\right)\sum_{n=0}^{\infty }\frac{q^{n}}{p^{n+1}}H\left(\frac{q^{n}}{p^{n+1}}\right).$$

**Lemma** **3.** [26] *The following equality is very import to prove ${\left(p,q\right)}_{\epsilon_{1}}$-integral:*

$$\int_{\epsilon_{1}}^{\epsilon_{2}}{\left(x-\epsilon_{1}\right)}^{\alpha }{\;}_{\epsilon_{1}}d_{p,q}x=\frac{{\left(\epsilon_{2}-\epsilon_{1}\right)}^{\alpha +1}}{{\left[\alpha +1\right]}_{p,q}},$$

*where α∈R\{−1}.*

## 4. Auxiliary Results

In order to obtain parameterized (p,q)-trapezoid and parameterized (p,q)-midpoint type integral inequalities through η-quasiconvex functions, we need following lemmas which we present in this section.

**Lemma** **4.** *Let $H:[\epsilon_{1},\epsilon_{2}]\to R$ be a (p,q)-differentiable function on $\left(\epsilon_{1},\epsilon_{2}\right)$ such that ${}_{\epsilon_{1}}D_{p,q}H$ is (p,q)-integrable on $\left[\epsilon_{1},\epsilon_{2}\right]$, then*

$$\begin{array}{cc} \; & p\mu H\left(\epsilon_{2}\right)+\left(1-p\mu \right)H\left(\epsilon_{1}\right)-\frac{1}{p(\epsilon_{2}-\epsilon_{1})}\int_{\epsilon_{1}}^{p\epsilon_{2}+\left(1-p\right)\epsilon_{1}}H\left(x\right)\;\epsilon_{1}d_{p,q}x\\ \; & =\left(\epsilon_{2}-\epsilon_{1}\right)\int_{0}^{1}\left(q\lambda +p\mu -1\right){\;}_{\epsilon_{1}}D_{p,q}H\left(\lambda \epsilon_{2}+\left(1-\lambda \right)\epsilon_{1}\right)\;\;d_{p,q}\lambda \end{array}$$

*for all λ,μ∈[0,1].*

**Proof.**  Consider

(12) $$\left(\epsilon_{2}-\epsilon_{1}\right)\int_{0}^{1}\left(q\lambda +p\mu -1\right){\;}_{\epsilon_{1}}D_{p,q}H\left(\lambda \epsilon_{2}+\left(1-\lambda \right)\epsilon_{1}\right)\;d_{p,q}\lambda$$

Applying Definitions 5 and 6, we have

(13) $$\begin{array}{cc} \; & \int_{0}^{1}\lambda \;{}_{\epsilon_{1}}D_{p,q}H\left(\lambda \epsilon_{2}+\left(1-\lambda \right)\epsilon_{1}\right)\;d_{p,q}\lambda \\ \; & =\int_{0}^{1}\frac{H\left(p\lambda \epsilon_{2}+\left(1-p\lambda \right)\epsilon_{1}\right)-H\left(q\lambda \epsilon_{2}+\left(1-q\lambda \right)\epsilon_{1}\right)}{\left(p-q\right)\left(\epsilon_{2}-\epsilon_{1}\right)}\;d_{p,q}\lambda \\ \; & =\frac{1}{\epsilon_{2}-\epsilon_{1}}\left[\sum_{n=0}^{\infty }\frac{q^{n}}{p^{n+1}}H\left(\frac{q^{n}}{p^{n}}\epsilon_{2}+\left(1-\frac{q^{n}}{p^{n}}\right)\epsilon_{1}\right)-\sum_{n=0}^{\infty }\frac{q^{n}}{p^{n+1}}H\left(\frac{q^{n+1}}{p^{n+1}}\epsilon_{2}+\left(1-\frac{q^{n+1}}{p^{n+1}}\right)\epsilon_{1}\right)\right]\\ \; & =\frac{1}{\epsilon_{2}-\epsilon_{1}}\left[\frac{1}{p}\sum_{n=0}^{\infty }\frac{q^{n}}{p^{n}}H\left(\frac{q^{n}}{p^{n}}\epsilon_{2}+\left(1-\frac{q^{n}}{p^{n}}\right)\epsilon_{1}\right)\right.\left.-\frac{1}{q}\sum_{n=1}^{\infty }\frac{q^{n}}{p^{n}}H\left(\frac{q^{n}}{p^{n}}\epsilon_{2}+\left(1-\frac{q^{n}}{p^{n}}\right)\epsilon_{1}\right)\right]\\ \; & =\frac{1}{\epsilon_{2}-\epsilon_{1}}\left[\frac{1}{q}H\left(\epsilon_{2}\right)-\left(\frac{1}{q}-\frac{1}{p}\right)\sum_{n=0}^{\infty }\frac{q^{n}}{p^{n}}H\left(\frac{q^{n}}{p^{n}}\epsilon_{2}+\left(1-\frac{q^{n}}{p^{n}}\right)\epsilon_{1}\right)\right]\\ \; & =\frac{1}{q\left(\epsilon_{2}-\epsilon_{1}\right)}H\left(\epsilon_{2}\right)-\frac{1}{pq{\left(\epsilon_{2}-\epsilon_{1}\right)}^{2}}\int_{\epsilon_{1}}^{p\epsilon_{2}+\left(1-p\right)\epsilon_{1}}H\left(x\right)\;{}_{\epsilon_{1}}d_{p,q}x \end{array}$$

and

(14) $$\begin{array}{cc} \; & \int_{0}^{1}{}_{\epsilon_{1}}D_{p,q}H\left(\lambda \epsilon_{2}+\left(1-\lambda \right)\epsilon_{1}\right)\;d_{p,q}\lambda \\ \; & =\int_{0}^{1}\frac{H\left(p\lambda \epsilon_{2}+\left(1-p\lambda \right)\epsilon_{1}\right)-H\left(q\lambda \epsilon_{2}+\left(1-q\lambda \right)\epsilon_{1}\right)}{\lambda \left(p-q\right)\left(\epsilon_{2}-\epsilon_{1}\right)}\;d_{p,q}\lambda \\ \; & =\frac{1}{\epsilon_{2}-\epsilon_{1}}\left[\sum_{n=0}^{\infty }H\left(\frac{q^{n}}{p^{n}}\epsilon_{2}+\left(1-\frac{q^{n}}{p^{n}}\right)\epsilon_{1}\right)-\sum_{n=0}^{\infty }H\left(\frac{q^{n+1}}{p^{n+1}}\epsilon_{2}+\left(1-\frac{q^{n+1}}{p^{n+1}}\right)\epsilon_{1}\right)\right]\\ \; & =\frac{H\left(\epsilon_{2}\right)-H\left(\epsilon_{1}\right)}{\epsilon_{2}-\epsilon_{1}}. \end{array}$$

Substituting (13) and (14) into (12), we obtain the desired result. □

**Lemma** **5.** *Let $H:[\epsilon_{1},\epsilon_{2}]\to R$ be a (p,q)-differentiable function on $\left(\epsilon_{1},\epsilon_{2}\right)$ such that ${}_{\epsilon_{1}}D_{p,q}H$ is a (p,q)-integrable on $\left[\epsilon_{1},\epsilon_{2}\right]$, then *

(15) $$\begin{array}{cc} \; & H\left(p\mu \epsilon_{2}+\left(1-p\mu \right)\epsilon_{1}\right)-\frac{1}{p(\epsilon_{2}-\epsilon_{1})}\int_{\epsilon_{1}}^{p\epsilon_{2}+\left(1-p\right)\epsilon_{1}}H\left(x\right)\;{}_{\epsilon_{1}}d_{p,q}x\\ \; & =\left(\epsilon_{2}-\epsilon_{1}\right)\left[\int_{0}^{p\mu }q\lambda {\;}_{\epsilon_{1}}D_{p,q}H\left(\lambda \epsilon_{2}+\left(1-\lambda \right)\epsilon_{1}\right)\;d_{p,q}\lambda +\int_{p\mu }^{1}\left(q\lambda -1\right){}_{\epsilon_{1}}D_{p,q}H\left(\lambda \epsilon_{2}+\left(1-\lambda \right)\epsilon_{1}\right)\;d_{p,q}\lambda \right] \end{array}$$

*for all λ,μ∈[0,1].*

**Proof.**  We are taking right part of equality (15),

$$\left(\epsilon_{2}-\epsilon_{1}\right)\left[\int_{0}^{p\mu }q\lambda \;{}_{\epsilon_{1}}D_{p,q}H\left(\lambda \epsilon_{2}+\left(1-\lambda \right)\epsilon_{1}\right)\;d_{p,q}\lambda +\int_{p\mu }^{1}\left(q\lambda -1\right)\;{}_{\epsilon_{1}}D_{p,q}H\left(\lambda \epsilon_{2}+\left(1-\lambda \right)\epsilon_{1}\right)\;d_{p,q}\lambda \right].$$

In this case, we are simplifying our integral by applying the identical transformation and get our required integral

(16) $$\begin{array}{cc} =(\epsilon_{2}-\epsilon_{1}) & \left[\int_{0}^{1}\left(q\lambda -1\right)\;{}_{\epsilon_{1}}D_{p,q}H\left(\lambda \epsilon_{2}+\left(1-\lambda \right)\epsilon_{1}\right)\;d_{p,q}\lambda +\int_{0}^{p\mu }{}_{\epsilon_{1}}D_{p,q}H\left(\lambda \epsilon_{2}+\left(1-\lambda \right)\epsilon_{1}\right)\;d_{p,q}\lambda \right]. \end{array}$$

Applying Definitions 5 and 6, we have

(17) $$\begin{array}{cc} \int_{0}^{1}\lambda {}_{\epsilon_{1}}D_{p,q}H\left(\lambda \epsilon_{2}+\left(1-\lambda \right)\epsilon_{1}\right)\;d_{p,q}\lambda & =\frac{1}{q\left(\epsilon_{2}-\epsilon_{1}\right)}H\left(\epsilon_{2}\right)-\frac{1}{pq{\left(\epsilon_{2}-\epsilon_{1}\right)}^{2}}\int_{\epsilon_{1}}^{p\epsilon_{2}+\left(1-p\right)\epsilon_{1}}H\left(x\right)\;{}_{\epsilon_{1}}d_{p,q}x \end{array}$$

(18) $$\begin{array}{cc} \int_{0}^{1}{}_{\epsilon_{1}}D_{p,q}H\left(\lambda \epsilon_{2}+\left(1-\lambda \right)\epsilon_{1}\right)\;d_{p,q}\lambda & =\frac{H\left(\epsilon_{2}\right)-H\left(\epsilon_{1}\right)}{\epsilon_{2}-\epsilon_{1}}, \end{array}$$

and

(19) $$\begin{array}{cc} \int_{0}^{p\mu }{}_{\epsilon_{1}}D_{p,q}H\left(\lambda \epsilon_{2}+\left(1-\lambda \right)\epsilon_{1}\right)\;d_{p,q}\lambda & =\int_{0}^{p\mu }\frac{H\left(p\lambda \epsilon_{2}+\left(1-p\lambda \right)\epsilon_{1}\right)-H\left(q\lambda \epsilon_{2}+\left(1-q\lambda \right)\epsilon_{1}\right)}{\lambda \left(p-q\right)\left(\epsilon_{2}-\epsilon_{1}\right)}\;d_{p,q}\lambda \\ \; & =\frac{1}{\epsilon_{2}-\epsilon_{1}}\left[\sum_{n=0}^{\infty }H\left(\frac{q^{n}}{p^{n}}p\mu \epsilon_{2}+\left(1-\frac{q^{n}}{p^{n}}p\mu \right)\epsilon_{1}\right)\right.\\ \; & \;-\left.\sum_{n=0}^{\infty }H\left(\frac{q^{n+1}}{p^{n+1}}p\mu \epsilon_{2}+\left(1-\frac{q^{n+1}}{p^{n+1}}p\mu \right)\epsilon_{1}\right)\right]\\ \; & =\frac{H\left(p\mu \epsilon_{2}+\left(1-p\mu \right)\epsilon_{1}\right)-H\left(\epsilon_{1}\right)}{\epsilon_{2}-\epsilon_{1}}. \end{array}$$

Substituting (17)–(19) into (16), we obtain the desired result. □

## 5. Main Results

**Theorem** **5.** *Let $H:[\epsilon_{1},\epsilon_{2}]\to R$ be a (p,q)-differentiable function on $\left(\epsilon_{1},\epsilon_{2}\right)$ and ${}_{\epsilon_{1}}D_{p,q}H$ be a (p,q)-integrable on $\left[\epsilon_{1},\epsilon_{2}\right]$ and $0\leq \epsilon_{1}<\epsilon_{2}<\infty$. If ${\left|{}_{\epsilon_{1}}D_{p,q}H\right|}^{\sigma }$ is an η-quasiconvex function on $\left[\epsilon_{1},\epsilon_{2}\right]$ for σ≥1. Then, the following inequality holds for all μ∈[0,1]*

(20) $$\begin{array}{cc} \; & \left|p\mu H\left(\epsilon_{2}\right)+\left(1-p\mu \right)H\left(\epsilon_{1}\right)-\frac{1}{p(\epsilon_{2}-\epsilon_{1})}\int_{\epsilon_{1}}^{p\epsilon_{2}+\left(1-p\right)\epsilon_{1}}H\left(x\right)\;{}_{\epsilon_{1}}d_{p,q}x\right|\\ & \leq \left(\epsilon_{2}-\epsilon_{1}\right)L\left(\mu ;p,q\right){\left(P_{\epsilon_{1}}^{\epsilon_{2}}\left(|{}_{\epsilon_{1}}D_{p,q}{H|}^{\sigma };\eta \right)\right)}^{\frac{1}{\sigma }} \end{array}$$

*where*

$$L\left(\mu ;p,q\right)=\left\{\begin{array}{cc} \frac{\left(p-p\mu \right){\left[2\right]}_{p,q}-qp^{2}}{{\left[2\right]}_{p,q}}, & \;for\;\;0\leq p\mu \leq 1-q;\\ \frac{2{\left(1-p\mu \right)}^{2}\left({\left[2\right]}_{p,q}-1\right)+p^{2}q^{2}-pq\left(1-p\mu \right){\left[2\right]}_{p,q}}{q{\left[2\right]}_{p,q}}, & \;for\;\;1-q<p\mu \leq 1. \end{array}\right.$$

**Proof.**  The η-quasiconvexity of ${\left|{}_{\epsilon_{1}}D_{p,q}H\right|}^{\sigma }$ on $\left[\epsilon_{1},\epsilon_{2}\right]$ such that for all λ∈[0,1], then

$$\begin{array}{cc} \; & \left(P_{\epsilon_{1}}^{\epsilon_{2}}\left(|{}_{\epsilon_{1}}D_{p,q}{H|}^{\sigma };\eta \right)\right)=:{\left|{}_{\epsilon_{1}}D_{p,q}H\left(\lambda \epsilon_{2}+\left(1-\lambda \right)\epsilon_{1}\right)\right|}^{\sigma }\\ & \leq \max\left\{{\left|{}_{\epsilon_{1}}D_{p,q}H(\epsilon_{1})\right|}^{\sigma },{\left|{}_{\epsilon_{1}}D_{p,q}H(\epsilon_{1})\right|}^{\sigma }+\eta \left({\left|{}_{\epsilon_{1}}D_{p,q}H(\epsilon_{2})\right|}^{\sigma },{\left|{}_{\epsilon_{1}}D_{p,q}H(\epsilon_{1})\right|}^{\sigma }\right)\right\}. \end{array}$$

From Lemma 4, utilizing the property of the modulus with Hölder’s inequality and using definition of η-quasiconvexity of $|{}_{\epsilon_{1}}D_{p,q}{H|}^{\sigma }$, we have

$$\begin{array}{cc} \; & \left|p\mu H\left(\epsilon_{2}\right)+\left(1-p\mu \right)H\left(\epsilon_{1}\right)-\frac{1}{p(\epsilon_{2}-\epsilon_{1})}\int_{\epsilon_{1}}^{p\epsilon_{2}+\left(1-p\right)\epsilon_{1}}H\left(x\right)\;{}_{\epsilon_{1}}d_{p,q}x\right|\\ \; & \leq \left(\epsilon_{2}-\epsilon_{1}\right)\int_{0}^{1}\left|q\lambda +p\mu -1\right|\left|{}_{\epsilon_{1}}D_{p,q}H\left(\lambda \epsilon_{2}+\left(1-\lambda \right)\epsilon_{1}\right)\right|\;d_{p,q}\lambda \\ \; & =\left(\epsilon_{2}-\epsilon_{1}\right)\int_{0}^{1}{\left|q\lambda +p\mu -1\right|}^{\frac{\sigma -1}{\sigma }}{\left|q\lambda +p\mu -1\right|}^{\frac{1}{\sigma }}\left|{}_{\epsilon_{1}}D_{p,q}H\left(\lambda \epsilon_{2}+\left(1-\lambda \right)\epsilon_{1}\right)\right|\;d_{p,q}\lambda \\ \; & \leq \left(\epsilon_{2}-\epsilon_{1}\right){\left(\int_{0}^{1}\left|q\lambda +p\mu -1\right|\;d_{p,q}\lambda \right)}^{\frac{\sigma -1}{\sigma }}{\left(\int_{0}^{1}\left|q\lambda +p\mu -1\right|{\left|{}_{\epsilon_{1}}D_{p,q}H\left(\lambda \epsilon_{2}+\left(1-\lambda \right)\epsilon_{1}\right)\right|}^{\sigma }\;d_{p,q}\lambda \right)}^{\frac{1}{\sigma }}\\ \; & \leq \left(\epsilon_{2}-\epsilon_{1}\right){\left(\int_{0}^{1}\left|q\lambda +p\mu -1\right|\;d_{p,q}\lambda \right)}^{\frac{\sigma -1}{\sigma }}{\left(\int_{0}^{1}\left|q\lambda +p\mu -1\right|\;d_{p,q}\lambda \right)}^{\frac{1}{\sigma }}{\left(P_{\epsilon_{1}}^{\epsilon_{2}}\left(|{}_{\epsilon_{1}}D_{p,q}{H|}^{\sigma };\eta \right)\right)}^{\frac{1}{\sigma }}\\ \; & =\left(\epsilon_{2}-\epsilon_{1}\right)\int_{0}^{1}\left|q\lambda +p\mu -1\right|\;d_{p,q}\lambda {\left(P_{\epsilon_{1}}^{\epsilon_{2}}\left(|{}_{\epsilon_{1}}D_{p,q}{H|}^{\sigma };\eta \right)\right)}^{\frac{1}{\sigma }}. \end{array}$$

Now,

$$L\left(\mu ;p,q\right)=\int_{0}^{1}\left|q\lambda +p\mu -1\right|\;d_{p,q}\lambda$$

$$=\left\{\begin{array}{cc} \frac{\left(p-p\mu \right){\left[2\right]}_{p,q}-qp^{2}}{{\left[2\right]}_{p,q}}, & \;\mathrm{for}\;\;0\leq p\mu \leq 1-q;\\ \frac{2{\left(1-p\mu \right)}^{2}\left({\left[2\right]}_{p,q}-1\right)+p^{2}q^{2}-pq\left(1-p\mu \right){\left[2\right]}_{p,q}}{q{\left[2\right]}_{p,q}}, & \;\mathrm{for}\;\;1-q<p\mu \leq 1. \end{array}\right.$$

This complete the proof. □

**Corollary** **1.** *Under the conditions of Theorem 5, the following inequality holds: *

(21) $$\begin{array}{cc} |p\mu H\left(\epsilon_{2}\right)+\left(1-p\mu \right)H\left(\epsilon_{1}\right) & -\frac{1}{p(\epsilon_{2}-\epsilon_{1})}\int_{\epsilon_{1}}^{p\epsilon_{2}+\left(1-p\right)\epsilon_{1}}H\left(x\right)\;{}_{\epsilon_{1}}d_{p,q}x|\\ \; & \leq \left(\epsilon_{2}-\epsilon_{1}\right)L\left(\mu ;p,q\right){\left[\max\left\{|{}_{\epsilon_{1}}D_{p,q}H(\epsilon_{1}){|}^{\sigma },{\left|{}_{\epsilon_{1}}D_{p,q}H(\epsilon_{2})\right|}^{\sigma }\right\}\right]}^{\frac{1}{\sigma }}, \end{array}$$

*where*

$$L\left(\mu ;p,q\right)=\left\{\begin{array}{cc} \frac{\left(p-p\mu \right){\left[2\right]}_{p,q}-qp^{2}}{{\left[2\right]}_{p,q}}, & \;for\;\;0\leq p\mu \leq 1-q;\\ \frac{2{\left(1-p\mu \right)}^{2}\left({\left[2\right]}_{p,q}-1\right)+p^{2}q^{2}-pq\left(1-p\mu \right){\left[2\right]}_{p,q}}{q{\left[2\right]}_{p,q}}, & \;for\;\;1-q<p\mu \leq 1. \end{array}\right.$$

**Remark** **5.** *If p=1, then* (20) *is reduced to the following:*

$$\left|\mu H\left(\epsilon_{2}\right)+\left(1-\mu \right)H\left(\epsilon_{1}\right)-\frac{1}{\epsilon_{2}-\epsilon_{1}}\int_{\epsilon_{1}}^{\epsilon_{2}}H\left(x\right)\;{}_{\epsilon_{1}}d_{q}x\right|\leq \left(\epsilon_{2}-\epsilon_{1}\right)L\left(\mu ;1,q\right){\left(P_{\epsilon_{1}}^{\epsilon_{2}}\left(|{}_{\epsilon_{1}}D_{q}{H|}^{\sigma };\eta \right)\right)}^{\frac{1}{\sigma }},$$

*where*

$$L\left(\mu ;1,q\right)=\left\{\begin{array}{cc} \frac{\left(1-\mu \right){\left[2\right]}_{q}-q}{{\left[2\right]}_{q}}, & \;\mathrm{for}\;\;0\leq \mu \leq 1-q;\\ \frac{2\mu^{2}+\mu \left(q-3\right)+1}{{\left[2\right]}_{q}}, & \;\mathrm{for}\;\;1-q<\mu \leq 1, \end{array}\right.$$

*which appeared in [15].*

**Remark** **6.** *If $\mu =\frac{1}{{\left[2\right]}_{p,q}}$ and $\eta \left(\epsilon_{2},\epsilon_{1}\right)=\epsilon_{2}-\epsilon_{1}$ in* (20)*, then Theorem 5 reduces to Theorem 5 proved in [21].*

**Remark** **7.** *If $\eta \left(\epsilon_{2},\epsilon_{1}\right)=\epsilon_{2}-\epsilon_{1}$ and $\mu =\frac{1}{2}$ for p=1 and $q\to 1^{-}$ in* (20)*, then Theorem 5 reduces to Theorem 6 proved in [35].*

**Theorem** **6.** *Let $H:[\epsilon_{1},\epsilon_{2}]\to R$ be a (p,q)-differentiable function on $\left(\epsilon_{1},\epsilon_{2}\right)$ and ${}_{\epsilon_{1}}D_{p,q}H$ be a (p,q)-integrable on $\left[\epsilon_{1},\epsilon_{2}\right]$ and $0\leq \epsilon_{1}<\epsilon_{2}<\infty$. If ${\left|{}_{\epsilon_{1}}D_{p,q}H\right|}^{\sigma_{2}}$ is an η-quasiconvex function on $\left[\epsilon_{1},\epsilon_{2}\right]$ for $\sigma_{2}>1$ with $\frac{1}{\sigma_{2}}+\frac{1}{\sigma_{1}}=1$, then the following inequality holds for all μ∈[0,1]:*

(22) $$\begin{array}{cc} |p\mu H\left(\epsilon_{2}\right)+\left(1-p\mu \right)H\left(\epsilon_{1}\right) & -\frac{1}{p(\epsilon_{2}-\epsilon_{1})}\int_{\epsilon_{1}}^{p\epsilon_{2}+\left(1-p\right)\epsilon_{1}}H\left(x\right)\;{}_{\epsilon_{1}}d_{p,q}x|\\ \; & \leq \left(\epsilon_{2}-\epsilon_{1}\right){\left[F_{q}^{\sigma_{1}}\left(p\mu ,\lambda \right)\right]}^{\frac{1}{\sigma_{1}}}{\left(P_{\epsilon_{1}}^{\epsilon_{2}}\left(|{}_{\epsilon_{1}}D_{p,q}{H|}^{\sigma_{2}};\eta \right)\right)}^{\frac{1}{\sigma_{2}}}, \end{array}$$

*where*

$$F_{q}^{\sigma_{1}}\left(p\mu ,\lambda \right)=\left\{\begin{array}{cc} \left(p-q\right)\sum_{n=0}^{\infty }\frac{q^{n}}{p^{n+1}}{\left(1-p\mu -\frac{q^{n+1}}{p^{n+1}}\right)}^{\sigma_{1}}, & \;for\;\;0\leq p\mu \leq 1-q;\\ \left(p-q\right){\left(1-p\mu \right)}^{\sigma_{1}+1}\sum_{n=0}^{\infty }\frac{q^{n-1}}{p^{n+1}}{\left(1-\frac{q^{n}}{p^{n+1}}\right)}^{\sigma_{1}} & \\ +\left(p-q\right)\sum_{n=0}^{\infty }\frac{q^{n}}{p^{n+1}}{\left(\frac{q^{n+1}}{p^{n+1}}-1+p\mu \right)}^{\sigma_{1}} & \\ -\left(p-q\right){\left(1-p\mu \right)}^{\sigma_{1}+1}\sum_{n=0}^{\infty }\frac{q^{n-1}}{p^{n+1}}{\left(\frac{q^{n}}{p^{n+1}}-1\right)}^{\sigma_{1}}, & \;for\;\;1-q<p\mu \leq 1. \end{array}\right.$$

**Proof.**  From Lemma 4, utilizing the property of the modulus with Hölder’s inequality and using definition of η-quasiconvexity of $|{}_{\epsilon_{1}}D_{p,q}{H|}^{\sigma_{2}}$ and λ∈[0,1], we have

$$\begin{array}{cc} \; & \left|p\mu H\left(\epsilon_{2}\right)+\left(1-p\mu \right)H\left(\epsilon_{1}\right)-\frac{1}{p(\epsilon_{2}-\epsilon_{1})}\int_{\epsilon_{1}}^{p\epsilon_{2}+\left(1-p\right)\epsilon_{1}}H\left(x\right)\;{}_{\epsilon_{1}}d_{p,q}x\right|\\ \; & \leq \left(\epsilon_{2}-\epsilon_{1}\right)\left[\int_{0}^{1}\left|q\lambda +p\mu -1\right|\left|{}_{\epsilon_{1}}D_{p,q}H\left(\lambda \epsilon_{2}+\left(1-\lambda \right)\epsilon_{1}\right)\right|\;d_{p,q}\lambda \right]\\ \; & \leq \left(\epsilon_{2}-\epsilon_{1}\right)\left[{\left(\int_{0}^{1}{\left|q\lambda +p\mu -1\right|}^{\sigma_{1}}\;d_{p,q}\lambda \right)}^{\frac{1}{\sigma_{1}}}{\left(\int_{0}^{1}{\left|{}_{\epsilon_{1}}D_{p,q}H\left(\lambda \epsilon_{2}+\left(1-\lambda \right)\epsilon_{1}\right)\right|}^{\sigma_{2}}\;d_{p,q}\lambda \right)}^{\frac{1}{\sigma_{2}}}\right]\\ \; & \leq \left(\epsilon_{2}-\epsilon_{1}\right)\left[{\left(\int_{0}^{1}{\left|q\lambda +p\mu -1\right|}^{\sigma_{1}}\;d_{p,q}\lambda \right)}^{\frac{1}{\sigma_{1}}}\left.{\left(P_{\epsilon_{1}}^{\epsilon_{2}}\left(|{}_{\epsilon_{1}}D_{p,q}{H|}^{\sigma_{2}};\eta \right)\right)}^{\frac{1}{\sigma_{2}}}\right]\right.. \end{array}$$

Now,

$$\begin{array}{cc} F_{q}^{\sigma_{1}}\left(p\mu ,\lambda \right) & =\int_{0}^{1}{\left|q\lambda +p\mu -1\right|}^{\sigma_{1}}\;d_{p,q}\lambda \\ \; & =\left\{\begin{array}{cc} \left(p-q\right)\sum_{n=0}^{\infty }\frac{q^{n}}{p^{n+1}}{\left(1-p\mu -\frac{q^{n+1}}{p^{n+1}}\right)}^{\sigma_{1}}, & \;\mathrm{for}\;\;0\leq p\mu \leq 1-q;\\ \left(p-q\right){\left(1-p\mu \right)}^{\sigma_{1}+1}\sum_{n=0}^{\infty }\frac{q^{n-1}}{p^{n+1}}{\left(1-\frac{q^{n}}{p^{n+1}}\right)}^{\sigma_{1}} & \\ +\left(p-q\right)\sum_{n=0}^{\infty }\frac{q^{n}}{p^{n+1}}{\left(\frac{q^{n+1}}{p^{n+1}}-1+p\mu \right)}^{\sigma_{1}} & \\ -\left(p-q\right){\left(1-p\mu \right)}^{\sigma_{1}+1}\sum_{n=0}^{\infty }\frac{q^{n-1}}{p^{n+1}}{\left(\frac{q^{n}}{p^{n+1}}-1\right)}^{\sigma_{1}}, & \;\mathrm{for}\;\;1-q<p\mu \leq 1. \end{array}\right. \end{array}$$

The proof is completed. □

**Corollary** **2.** *Under the conditions of Theorem 6, the following inequalities are true:*

*(i)* 
(23) $$\begin{array}{cc} \; & \left|p\mu H\left(\epsilon_{2}\right)+\left(1-p\mu \right)H\left(\epsilon_{1}\right)-\frac{1}{p(\epsilon_{2}-\epsilon_{1})}\int_{\epsilon_{1}}^{p\epsilon_{2}+\left(1-p\right)\epsilon_{1}}H\left(x\right)\;{}_{\epsilon_{1}}d_{p,q}x\right|\\ \; & \leq \left(\epsilon_{2}-\epsilon_{1}\right){\left[F_{q}^{\sigma_{1}}\left(p\mu ,\lambda \right)\right]}^{\frac{1}{\sigma_{1}}}{\left[\max\left\{|{}_{\epsilon_{1}}D_{p,q}H(\epsilon_{1}){|}^{\sigma_{2}},{\left|{}_{\epsilon_{1}}D_{p,q}H(\epsilon_{2})\right|}^{\sigma_{2}}\right\}\right]}^{\frac{1}{\sigma_{2}}}, \end{array}$$
  *where*
$$F_{q}^{\sigma_{1}}\left(p\mu ,\lambda \right)=\left\{\begin{array}{cc} \left(p-q\right)\sum_{n=0}^{\infty }\frac{q^{n}}{p^{n+1}}{\left(1-p\mu -\frac{q^{n+1}}{p^{n+1}}\right)}^{\sigma_{1}}, & \;\mathrm{for}\;\;0\leq p\mu \leq 1-q;\\ \left(p-q\right){\left(1-p\mu \right)}^{\sigma_{1}+1}\sum_{n=0}^{\infty }\frac{q^{n-1}}{p^{n+1}}{\left(1-\frac{q^{n}}{p^{n+1}}\right)}^{\sigma_{1}} & \\ +\left(p-q\right)\sum_{n=0}^{\infty }\frac{q^{n}}{p^{n+1}}{\left(\frac{q^{n+1}}{p^{n+1}}-1+p\mu \right)}^{\sigma_{1}} & \\ -\left(p-q\right){\left(1-p\mu \right)}^{\sigma_{1}+1}\sum_{n=0}^{\infty }\frac{q^{n-1}}{p^{n+1}}{\left(\frac{q^{n}}{p^{n+1}}-1\right)}^{\sigma_{1}}, & \;\mathrm{for}\;\;1-q<p\mu \leq 1. \end{array}\right.$$
*(ii)* 
(24) $$\begin{array}{cc} \; & \left|\frac{pH\left(\epsilon_{2}\right)+qH\left(\epsilon_{1}\right)}{2}-\frac{1}{p(\epsilon_{2}-\epsilon_{1})}\int_{\epsilon_{1}}^{p\epsilon_{2}+\left(1-p\right)\epsilon_{1}}H\left(x\right)\;{}_{\epsilon_{1}}d_{p,q}x\right|\\ \; & \leq \left(\epsilon_{2}-\epsilon_{1}\right){\left[F_{q}^{\sigma_{1}}\left(\frac{p}{2},\lambda \right)\right]}^{\frac{1}{\sigma_{1}}}{\left[\max\left\{|{}_{\epsilon_{1}}D_{p,q}H(\epsilon_{1}){|}^{\sigma_{2}},{\left|{}_{\epsilon_{1}}D_{p,q}H(\epsilon_{2})\right|}^{\sigma_{2}}\right\}\right]}^{\frac{1}{\sigma_{2}}}. \end{array}$$

**Remark** **8.** *If $\eta \left(\epsilon_{2},\epsilon_{1}\right)=\epsilon_{2}-\epsilon_{1}$ in* (22)*, then Theorem 6 reduces to Theorem 6 proved in [21].*

**Remark** **9.** *If p=1 in* (22)*, then Theorem 6 reduces to Theorem 18 proved in [15].*

**Theorem** **7.** *Let $H:[\epsilon_{1},\epsilon_{2}]\to R$ be a (p,q)-differentiable function on $\left(\epsilon_{1},\epsilon_{2}\right)$ and ${}_{\epsilon_{1}}D_{p,q}H$ be a (p,q)-integrable on $\left[\epsilon_{1},\epsilon_{2}\right]$ and $0\leq \epsilon_{1}<\epsilon_{2}<\infty$. If ${\left|{}_{\epsilon_{1}}D_{p,q}H\right|}^{\sigma }$ is an η-quasiconvex function on $\left[\epsilon_{1},\epsilon_{2}\right]$ for σ≥1. Then, the following inequality holds for all μ∈[0,1]: *

(25) $$\begin{array}{cc} \left|H(p\mu \epsilon_{2}+\left(1-p\mu \right)\epsilon_{1}\right) & -\frac{1}{p(\epsilon_{2}-\epsilon_{1})}\int_{\epsilon_{1}}^{p\epsilon_{2}+\left(1-p\right)\epsilon_{1}}H\left(x\right)\;{}_{\epsilon_{1}}d_{p,q}x|\\ \; & \leq \frac{\left(\epsilon_{2}-\epsilon_{1}\right)\left[2qp^{2}\mu^{2}-q+\left(1-p\mu \right){\left[2\right]}_{p,q}\right]}{{\left[2\right]}_{p,q}}{\left[P_{\epsilon_{1}}^{\epsilon_{2}}\left(|{}_{\epsilon_{1}}D_{p,q}{H|}^{\sigma };\eta \right)\right]}^{\frac{1}{\sigma }}. \end{array}$$

**Proof.**  From Lemma 5, utilizing the property of the modulus with Hölder’s inequality and using definition of η-quasiconvexity of $|{}_{\epsilon_{1}}D_{p,q}{H|}^{\sigma }$ and λ∈[0,1], we have

$$\begin{array}{cc} \; & \left|H\left(p\mu \epsilon_{2}+\left(1-p\mu \right)\epsilon_{1}\right)-\frac{1}{p(\epsilon_{2}-\epsilon_{1})}\int_{\epsilon_{1}}^{p\epsilon_{2}+\left(1-p\right)\epsilon_{1}}H\left(x\right)\;{}_{\epsilon_{1}}d_{p,q}x\right|\\ \; & \leq \left(\epsilon_{2}-\epsilon_{1}\right)\left[\int_{0}^{p\mu }q\lambda \left|{\;}_{\epsilon_{1}}D_{p,q}H\left(\lambda \epsilon_{2}+\left(1-\lambda \right)\epsilon_{1}\right)\right|\;d_{p,q}\lambda \right.\\ \; & \left.+\int_{p\mu }^{1}\left|q\lambda -1\right|\left|\;{}_{\epsilon_{1}}D_{p,q}H\left(\lambda \epsilon_{2}+\left(1-\lambda \right)\epsilon_{1}\right)\right|\;d_{p,q}\lambda \right]\\ \; & \leq \left(\epsilon_{2}-\epsilon_{1}\right)\left[q{\left(\int_{0}^{p\mu }\lambda \;d_{p,q}\lambda \right)}^{\frac{\sigma -1}{\sigma }}{\left(\int_{0}^{p\mu }\lambda {\left|{}_{\epsilon_{1}}D_{p,q}H\left(\lambda \epsilon_{2}+\left(1-\lambda \right)\epsilon_{1}\right)\right|}^{\sigma }\;d_{p,q}\lambda \right)}^{\frac{1}{\sigma }}\right.\\ \; & \;+\left.{\left(\int_{p\mu }^{1}\left|q\lambda -1\right|\;d_{p,q}\lambda \right)}^{\frac{\sigma -1}{\sigma }}\left(\int_{p\mu }^{1}{\left|q\lambda -1{\left|{}_{\epsilon_{1}}D_{p,q}H\left(\lambda \epsilon_{2}+\left(1-\lambda \right)\epsilon_{1}\right)\right|}^{\sigma }\;d_{p,q}\lambda \right)}^{\frac{1}{\sigma }}\right]\right. \end{array}$$

(26) $$\begin{array}{cc} \; & \leq \left(\epsilon_{2}-\epsilon_{1}\right)\left[q{\left(\int_{0}^{p\mu }\lambda \;d_{p,q}\lambda \right)}^{\frac{\sigma -1}{\sigma }}{\left(\int_{0}^{p\mu }\lambda \;d_{p,q}\lambda \right)}^{\frac{1}{\sigma }}\right.\\ \; & \;+{\left(\int_{p\mu }^{1}\left|q\lambda -1\right|\;d_{p,q}\lambda \right)}^{\frac{\sigma -1}{\sigma }}\left.{\left(\int_{p\mu }^{1}\left|q\lambda -1\right|\;d_{p,q}\lambda \right)}^{\frac{1}{\sigma }}\right]{\left[P_{\epsilon_{1}}^{\epsilon_{2}}\left(|{}_{\epsilon_{1}}D_{p,q}{H|}^{\sigma };\eta \right)\right]}^{\frac{1}{\sigma }}\\ \; & =\left(\epsilon_{2}-\epsilon_{1}\right)\left[q\int_{0}^{p\mu }\lambda \;d_{p,q}\lambda +\int_{p\mu }^{1}\left|q\lambda -1\right|\;d_{p,q}\lambda \right]{\left[P_{\epsilon_{1}}^{\epsilon_{2}}\left(|{}_{\epsilon_{1}}D_{p,q}{H|}^{\sigma };\eta \right)\right]}^{\frac{1}{\sigma }}. \end{array}$$

Now, using Definition 6, we get that

(27) $$\int_{0}^{p\mu }\lambda \;d_{p,q}\lambda =\frac{p^{2}\mu^{2}}{{\left[2\right]}_{p,q}}.$$

Also, using fact that qλ<1, we obtain that

(28) $$\begin{array}{cc} \int_{p\mu }^{1}\left|q\lambda -1\right|\;d_{p,q}\lambda & =\int_{p\mu }^{1}\left(1-q\lambda \right)\;d_{p,q}\lambda \\ \; & =\int_{0}^{1}\left(1-q\lambda \right)\;d_{p,q}\lambda -\int_{0}^{p\mu }\left(1-q\lambda \right)\;d_{p,q}\lambda \\ \; & =\int_{0}^{1}1\;d_{p,q}\lambda -q\int_{0}^{1}\lambda \;d_{p,q}\lambda -\int_{0}^{p\mu }1\;d_{p,q}\lambda +q\int_{0}^{p\mu }\lambda \;d_{p,q}\lambda \\ \; & =1-\frac{q}{{\left[2\right]}_{p,q}}-p\mu +\frac{qp^{2}\mu^{2}-q+\left(1-p\mu \right){\left[2\right]}_{p,q}}{{\left[2\right]}_{p,q}}. \end{array}$$

We get the intended result by combining (26)–(28). □

**Remark** **10.** *If we take p=1 in Theorem 7, then we recovered Theorem 20, which is proved in [15].*

**Corollary** **3.** *Under the conditions of Theorem 7, the following inequalities are true:*

*(i)* 
(29) $$\left|H\left(\epsilon_{1}\right)-\frac{1}{p(\epsilon_{2}-\epsilon_{1})}\int_{\epsilon_{1}}^{p\epsilon_{2}+\left(1-p\right)\epsilon_{1}}H\left(x\right){\;}_{\epsilon_{1}}d_{p,q}x\right|\leq \frac{\left(\epsilon_{2}-\epsilon_{1}\right)\left({\left[2\right]}_{p,q}-q\right)}{{\left[2\right]}_{p,q}}{\left[P_{\epsilon_{1}}^{\epsilon_{2}}\left(|{}_{\epsilon_{1}}D_{p,q}{H|}^{\sigma };\eta \right)\right]}^{\frac{1}{\sigma }}.$$
*(ii)* 
(30) $$\begin{array}{cc} \left|H(\epsilon_{2}+\left(1-p\right)\epsilon_{1}\right) & -\frac{1}{p(\epsilon_{2}-\epsilon_{1})}\int_{\epsilon_{1}}^{p\epsilon_{2}+\left(1-p\right)\epsilon_{1}}H\left(x\right)\;{}_{\epsilon_{1}}d_{p,q}x|\\ \; & \leq \frac{\left(\epsilon_{2}-\epsilon_{1}\right)\left(2qp^{2}-q+\left(1-p\right){\left[2\right]}_{p,q}\right)}{{\left[2\right]}_{p,q}}{\left[P_{\epsilon_{1}}^{\epsilon_{2}}\left(|{}_{\epsilon_{1}}D_{p,q}{H|}^{\sigma };\eta \right)\right]}^{\frac{1}{\sigma }}. \end{array}$$
*(iii)* 
(31) $$\begin{array}{cc} |H\left(\frac{p\epsilon_{2}+\left(2-p\right)\epsilon_{1}}{2}\right) & -\frac{1}{p(\epsilon_{2}-\epsilon_{1})}\int_{\epsilon_{1}}^{p\epsilon_{2}+\left(1-p\right)\epsilon_{1}}H\left(x\right)\;{}_{\epsilon_{1}}d_{p,q}x|\\ \; & \leq \frac{\left(\epsilon_{2}-\epsilon_{1}\right)\left(qp^{2}-2q+\left(2-p\right){\left[2\right]}_{p,q}\right)}{2{\left[2\right]}_{p,q}}{\left[P_{\epsilon_{1}}^{\epsilon_{2}}\left(|{}_{\epsilon_{1}}D_{p,q}{H|}^{\sigma };\eta \right)\right]}^{\frac{1}{\sigma }}. \end{array}$$
*(iv)* 
(32) $$\begin{array}{cc} |H\left(\frac{p\epsilon_{2}+q\epsilon_{1}}{{\left[2\right]}_{p,q}}\right) & -\frac{1}{p(\epsilon_{2}-\epsilon_{1})}\int_{\epsilon_{1}}^{p\epsilon_{2}+\left(1-p\right)\epsilon_{1}}H\left(x\right)\;{}_{\epsilon_{1}}d_{p,q}x|\leq \frac{\left(\epsilon_{2}-\epsilon_{1}\right)2qp^{2}}{{\left[2\right]}_{p,q}^{3}}{\left[P_{\epsilon_{1}}^{\epsilon_{2}}\left(|{}_{\epsilon_{1}}D_{p,q}{H|}^{\sigma };\eta \right)\right]}^{\frac{1}{\sigma }}. \end{array}$$

**Remark** **11.** *If $\eta \left(\epsilon_{2},\epsilon_{1}\right)=\epsilon_{2}-\epsilon_{1}$, then* (32) *reduces to*

(33) $$\begin{array}{cc} |H\left(\frac{p\epsilon_{2}+q\epsilon_{1}}{{\left[2\right]}_{p,q}}\right) & -\frac{1}{p(\epsilon_{2}-\epsilon_{1})}\int_{\epsilon_{1}}^{p\epsilon_{2}+\left(1-p\right)\epsilon_{1}}H\left(x\right)\;{}_{\epsilon_{1}}d_{p,q}x|\\ \; & \leq \frac{\left(\epsilon_{2}-\epsilon_{1}\right)2qp^{2}}{{\left[2\right]}_{p,q}^{3}}{\left[\max\left(|{}_{\epsilon_{1}}D_{p,q}H\left(\epsilon_{1}\right){|}^{\sigma_{2}},{\left|{}_{\epsilon_{1}}D_{p,q}H\left(\epsilon_{2}\right)\right|}^{\sigma_{2}}\right)\right]}^{\frac{1}{\sigma_{2}}}, \end{array}$$

*which appeared in [20].*

**Theorem** **8.** *Let $H:[\epsilon_{1},\epsilon_{2}]\to R$ be a (p,q)-differentiable function on $\left(\epsilon_{1},\epsilon_{2}\right)$ and ${}_{\epsilon_{1}}D_{p,q}H$ be a (p,q)-integrable on $\left[\epsilon_{1},\epsilon_{2}\right]$ and $0\leq \epsilon_{1}<\epsilon_{2}<\infty$. If ${\left|{}_{\epsilon_{1}}D_{p,q}H\right|}^{\sigma_{2}}$ is a η-quasiconvex function on $\left[\epsilon_{1},\epsilon_{2}\right]$ for $\sigma_{2}>1$ with $\frac{1}{\sigma_{2}}+\frac{1}{\sigma_{1}}=1.$ Then the following inequality holds for all μ∈[0,1]: *

(34) $$\begin{array}{cc} \; & \left|H\left(p\mu \epsilon_{2}+\left(1-p\mu \right)\epsilon_{1}\right)-\frac{1}{p(\epsilon_{2}-\epsilon_{1})}\int_{\epsilon_{1}}^{p\epsilon_{2}+\left(1-p\right)\epsilon_{1}}H\left(x\right)\;{}_{\epsilon_{1}}d_{p,q}x\right|\\ \; & \leq \left(\epsilon_{2}-\epsilon_{1}\right)\left[q{\left(\frac{{\left(p\mu \right)}^{\sigma_{1}+1}\left(p-q\right)}{p^{\sigma_{1}+1}-q^{\sigma_{1}+1}}\right)}^{\frac{1}{\sigma_{1}}}{\left(p\mu \right)}^{\frac{1}{\sigma_{2}}}+{\left(Q_{p,q}\left(p\mu ;\sigma_{1}\right)\right)}^{\frac{1}{\sigma_{1}}}{\left(1-p\mu \right)}^{\frac{1}{\sigma_{2}}}\right]{\left(P_{\epsilon_{1}}^{\epsilon_{2}}\left(|{}_{\epsilon_{1}}D_{p,q}{H|}^{\sigma_{2}};\eta \right)\right)}^{\frac{1}{\sigma_{2}}}, \end{array}$$

*where*

$$Q_{p,q}\left(p\mu ,\sigma_{1}\right)=\int_{p\mu }^{1}{\left|q\lambda -1\right|}^{\sigma_{1}}\;d_{p,q}\lambda .$$

**Proof.**  From Lemma 5, utilizing the property of the modulus with Hölder’s inequality and using definition of η-quasiconvexity of $|{}_{\epsilon_{1}}D_{p,q}{H|}^{\sigma_{2}}$ and λ∈[0,1], we have

(35) $$\begin{array}{cc} \; & \left|H\left(p\mu \epsilon_{2}+\left(1-p\mu \right)\epsilon_{1}\right)-\frac{1}{p(\epsilon_{2}-\epsilon_{1})}\int_{\epsilon_{1}}^{p\epsilon_{2}+\left(1-p\right)\epsilon_{1}}H\left(x\right)\;{}_{\epsilon_{1}}d_{p,q}x\right|\\ \; & \leq \left(\epsilon_{2}-\epsilon_{1}\right)\left[\int_{0}^{p\mu }q\lambda \left|{\;}_{\epsilon_{1}}D_{p,q}H\left(\lambda \epsilon_{2}+\left(1-\lambda \right)\epsilon_{1}\right)\right|\;d_{p,q}\lambda +\int_{p\mu }^{1}\left|q\lambda -1\right|\left|\;{}_{\epsilon_{1}}D_{p,q}H\left(\lambda \epsilon_{2}+\left(1-\lambda \right)\epsilon_{1}\right)\right|\;d_{p,q}\lambda \right]\\ \; & \leq \left(\epsilon_{2}-\epsilon_{1}\right)\left[q{\left(\int_{0}^{p\mu }\lambda^{\sigma_{1}}\;d_{p,q}\lambda \right)}^{\frac{1}{\sigma_{1}}}{\left(\int_{0}^{p\mu }{\left|{}_{\epsilon_{1}}D_{p,q}H\left(\lambda \epsilon_{2}+\left(1-\lambda \right)\epsilon_{1}\right)\right|}^{\sigma_{2}}\;d_{p,q}\lambda \right)}^{\frac{1}{\sigma_{2}}}\right.\\ \; & \;+\left.{\left(\int_{p\mu }^{1}{\left|q\lambda -1\right|}^{\sigma_{1}}\;d_{p,q}\lambda \right)}^{\frac{1}{\sigma_{1}}}\left(\int_{p\mu }^{1}{\left.{\left|{}_{\epsilon_{1}}D_{p,q}H\left(\lambda \epsilon_{2}+\left(1-\lambda \right)\epsilon_{1}\right)\right|}^{\sigma_{2}}\;d_{p,q}\lambda \right)}^{\frac{1}{\sigma_{2}}}\right]\right.\\ \; & \leq \left(\epsilon_{2}-\epsilon_{1}\right)\left[q{\left(\int_{0}^{p\mu }\lambda^{\sigma_{1}}\;d_{p,q}\lambda \right)}^{\frac{1}{\sigma_{1}}}{\left(P_{\epsilon_{1}}^{\epsilon_{2}}\left(|{}_{\epsilon_{1}}D_{p,q}{H|}^{\sigma_{2}};\eta \right)\int_{0}^{p\mu }1\;d_{p,q}\lambda \right)}^{\frac{1}{\sigma_{2}}}\right.\\ \; & \;+\left.{\left(\int_{p\mu }^{1}{\left|q\lambda -1\right|}^{\sigma_{1}}\;d_{p,q}\lambda \right)}^{\frac{1}{\sigma_{1}}}\left(P_{\epsilon_{1}}^{\epsilon_{2}}\left(|{}_{\epsilon_{1}}D_{p,q}{H|}^{\sigma_{2}};\eta \right)\int_{p\mu }^{1}{\left.1\;d_{p,q}\lambda \right)}^{\frac{1}{\sigma_{2}}}\right]\right.. \end{array}$$

From Definition 6, we deduce that

$$\begin{array}{cc} \int_{0}^{p\mu }1\;d_{p,q}\lambda & =p\mu \\ \int_{p\mu }^{1}1\;d_{p,q}\lambda & =1-p\mu .\\ \int_{0}^{p\mu }\lambda^{\sigma_{1}}\;d_{p,q}\lambda & =\frac{{\left(p\mu \right)}^{\sigma_{1}+1}\left(p-q\right)}{p^{\sigma_{1}+1}-q^{\sigma_{1}+1}}\\ Q\left(p\mu ;\sigma_{1}\right) & =\int_{p\mu }^{1}{\left|q\lambda -1\right|}^{\sigma_{1}}\;d_{p,q}\lambda . \end{array}$$

Therefore, the proof is completed. □

**Corollary** **4.** *Under the conditions of Theorem 8, the following inequalities are true:*

*(i)* 
(36) $$\left|H\left(\epsilon_{1}\right)-\frac{1}{p(\epsilon_{2}-\epsilon_{1})}\int_{\epsilon_{1}}^{p\epsilon_{2}+\left(1-p\right)\epsilon_{1}}H\left(x\right)\;{}_{\epsilon_{1}}d_{p,q}x\right|\leq \left(\epsilon_{2}-\epsilon_{1}\right){\left(Q\left(0;\sigma_{1}\right)\right)}^{\frac{1}{\sigma_{1}}}{\left(P_{\epsilon_{1}}^{\epsilon_{2}}\left(|{}_{\epsilon_{1}}D_{p,q}{H|}^{\sigma_{2}};\eta \right)\right)}^{\frac{1}{\sigma_{2}}}.$$
*(ii)* 
(37) $$\begin{array}{cc} \; & \left|H\left(p\epsilon_{2}+\left(1-p\right)\epsilon_{1}\right)-\frac{1}{p(\epsilon_{2}-\epsilon_{1})}\int_{\epsilon_{1}}^{p\epsilon_{2}+\left(1-p\right)\epsilon_{1}}H\left(x\right)\;{}_{\epsilon_{1}}d_{p,q}x\right|\\ \; & \leq \left(\epsilon_{2}-\epsilon_{1}\right)\left[q{\left(\frac{p^{\sigma_{1}+1}\left(p-q\right)}{p^{\sigma_{1}+1}-q^{\sigma_{1}+1}}\right)}^{\frac{1}{\sigma_{1}}}p^{\frac{1}{\sigma_{2}}}+{\left(Q\left(p;\sigma_{1}\right)\right)}^{\frac{1}{\sigma_{1}}}{\left(1-p\right)}^{\frac{1}{\sigma_{2}}}\right]{\left(P_{\epsilon_{1}}^{\epsilon_{2}}\left(|{}_{\epsilon_{1}}D_{p,q}{H|}^{\sigma_{2}};\eta \right)\right)}^{\frac{1}{\sigma_{2}}}. \end{array}$$
*(iii)* 
(38) $$\begin{array}{cc} \; & \left|H\left(\frac{p\epsilon_{2}+\left(1-p\right)\epsilon_{1}}{2}\right)-\frac{1}{p(\epsilon_{2}-\epsilon_{1})}\int_{\epsilon_{1}}^{p\epsilon_{2}+\left(1-p\right)\epsilon_{1}}H\left(x\right)\;{}_{\epsilon_{1}}d_{p,q}x\right|\\ \; & \leq \frac{\left(\epsilon_{2}-\epsilon_{1}\right)}{2^{\frac{1}{\sigma_{2}}}}\left[q{\left(\frac{p^{\sigma_{1}+1}\left(p-q\right)}{2^{\sigma_{1}+1}\left(p^{\sigma_{1}+1}-q^{\sigma_{1}+1}\right)}\right)}^{\frac{1}{\sigma_{1}}}p^{\frac{1}{\sigma_{2}}}+{\left(Q\left(\frac{p}{2};\sigma_{1}\right)\right)}^{\frac{1}{\sigma_{1}}}{\left(2-p\right)}^{\frac{1}{\sigma_{2}}}\right]{\left(P_{\epsilon_{1}}^{\epsilon_{2}}\left(|{}_{\epsilon_{1}}D_{p,q}{H|}^{\sigma_{2}};\eta \right)\right)}^{\frac{1}{\sigma_{2}}}. \end{array}$$
*(iv)* 
(39) $$\begin{array}{cc} \; & \left|H\left(\frac{p\epsilon_{2}+q\epsilon_{1}}{{\left[2\right]}_{p,q}}\right)-\frac{1}{p(\epsilon_{2}-\epsilon_{1})}\int_{\epsilon_{1}}^{p\epsilon_{2}+\left(1-p\right)\epsilon_{1}}H\left(x\right)\;{}_{\epsilon_{1}}d_{p,q}x\right|\leq {\left(P_{\epsilon_{1}}^{\epsilon_{2}}\left(|{}_{\epsilon_{1}}D_{p,q}{H|}^{\sigma_{2}};\eta \right)\right)}^{\frac{1}{\sigma_{2}}}\\ \; & \;\times \frac{\left(\epsilon_{2}-\epsilon_{1}\right)}{{\left[2\right]}_{p,q}^{\frac{1}{\sigma_{2}}}}\left[q{\left(\frac{p^{\sigma_{1}+1}\left(p-q\right)}{{\left[2\right]}_{p,q}^{\sigma_{1}+1}\left(p^{\sigma_{1}+1}-q^{\sigma_{1}+1}\right)}\right)}^{\frac{1}{\sigma_{1}}}p^{\frac{1}{\sigma_{2}}}+{\left(Q\left(\frac{p}{{\left[2\right]}_{p,q}};\sigma_{1}\right)\right)}^{\frac{1}{\sigma_{1}}}{\left({\left[2\right]}_{p,q}-p\right)}^{\frac{1}{\sigma_{2}}}\right]. \end{array}$$

**Remark** **12.** *If $\eta \left(\epsilon_{2},\epsilon_{1}\right)=\epsilon_{2}-\epsilon_{1}$, then* (39) *reduces to*

(40) $$\begin{array}{cc} \; & \left|H\left(\frac{p\epsilon_{2}+q\epsilon_{1}}{{\left[2\right]}_{p,q}}\right)-\frac{1}{p(\epsilon_{2}-\epsilon_{1})}\int_{\epsilon_{1}}^{p\epsilon_{2}+\left(1-p\right)\epsilon_{1}}H\left(x\right)\;{}_{\epsilon_{1}}d_{p,q}x\right|\leq {\left[\max\left(|{}_{\epsilon_{1}}D_{p,q}H\left(\epsilon_{1}\right){|}^{\sigma_{2}},{\left|{}_{\epsilon_{1}}D_{p,q}H\left(\epsilon_{2}\right)\right|}^{\sigma_{2}}\right)\right]}^{\frac{1}{\sigma_{2}}}\\ \; & \;\times \frac{\left(\epsilon_{2}-\epsilon_{1}\right)}{{\left[2\right]}_{p,q}^{\frac{1}{\sigma_{2}}}}\left[q{\left(\frac{p^{\sigma_{1}+1}\left(p-q\right)}{{\left[2\right]}_{p,q}^{\sigma_{1}+1}\left(p^{\sigma_{1}+1}-q^{\sigma_{1}+1}\right)}\right)}^{\frac{1}{\sigma_{1}}}p^{\frac{1}{\sigma_{2}}}+{\left(Q\left(\frac{p}{{\left[2\right]}_{p,q}};\sigma_{1}\right)\right)}^{\frac{1}{\sigma_{1}}}{\left({\left[2\right]}_{p,q}-p\right)}^{\frac{1}{\sigma_{2}}}\right], \end{array}$$

*which appeared in [20].*

## 6. Application to Special Means

The special means for positive real numbers would be used as follows:

1. Arithmetic mean
  $$A\left(\epsilon_{1},\epsilon_{2}\right)=\frac{\epsilon_{1}+\epsilon_{2}}{2}.$$
2. Generalized logarithmic mean
  $$L_{k}\left(\epsilon_{1},\epsilon_{2}\right)={\left(\frac{\epsilon_{2}^{k+1}-\epsilon_{1}^{k+1}}{\left(k+1\right)\left(\epsilon_{2}-\epsilon_{1}\right)}\right)}^{\frac{1}{k}},\;k\in R\backslash \left\{-1,0\right\}.$$

**Proposition** **1.** *Suppose that $\epsilon_{1},\epsilon_{2}$ are two positive real numbers such that $\epsilon_{1}<\epsilon_{2}$ and 0<q<p≤1, then *

(41) $$\begin{array}{cc} \; & \left|\frac{q\epsilon_{1}^{2}+p\epsilon_{2}^{2}}{{\left[2\right]}_{p,q}}-\frac{p\epsilon_{1}^{2}\left\{p{\left[2\right]}_{p,q}+{\left[3\right]}_{p,q}\left[{\left[2\right]}_{p,q}-2\right]\right\}+2p\epsilon_{1}\epsilon_{2}\left[{\left[3\right]}_{p,q}-p{\left[2\right]}_{p,q}\right]+p^{2}\epsilon_{2}^{2}{\left[2\right]}_{p,q}}{{\left[2\right]}_{p,q}{\left[3\right]}_{p,q}}\right|\\ \; & \leq \frac{\left(\epsilon_{2}-\epsilon_{1}\right)2qp^{2}}{{\left[2\right]}_{p,q}^{3}}\max\left\{2\epsilon_{1},\left(2-{\left[2\right]}_{p,q}\right)\epsilon_{1}+{\left[2\right]}_{p,q}\epsilon_{2}\right\}. \end{array}$$

**Proof.**  Let $H\left(\kappa \right)=\kappa^{2}$. Then, we have

$$\begin{array}{cc} \; & \int_{\epsilon_{1}}^{p\epsilon_{2}+\left(1-p\right)\epsilon_{1}}\kappa^{2}{\;}_{\epsilon_{1}}d_{p,q}\kappa =\int_{\epsilon_{1}}^{p\epsilon_{2}+\left(1-p\right)\epsilon_{1}}{\left(\kappa -\epsilon_{1}+\epsilon_{1}\right)}^{2}{\;}_{\epsilon_{1}}d_{p,q}\kappa \\ \; & =\int_{\epsilon_{1}}^{p\epsilon_{2}+\left(1-p\right)\epsilon_{1}}{\left(\kappa -\epsilon_{1}\right)}^{2}{\;}_{\epsilon_{1}}d_{p,q}\kappa +2\epsilon_{1}\int_{\epsilon_{1}}^{p\epsilon_{2}+\left(1-p\right)\epsilon_{1}}\left(\kappa -\epsilon_{1}\right){\;}_{\epsilon_{1}}d_{p,q}\kappa +\epsilon_{1}^{2}\int_{\epsilon_{1}}^{p\epsilon_{2}+\left(1-p\right)\epsilon_{1}}1{\;}_{\epsilon_{1}}d_{p,q}\kappa \\ \; & =\frac{p^{3}{\left(\epsilon_{2}-\epsilon_{1}\right)}^{3}}{{\left[3\right]}_{p,q}}+2\epsilon_{1}\frac{p^{2}{\left(\epsilon_{2}-\epsilon_{1}\right)}^{2}}{{\left[2\right]}_{p,q}}+\epsilon_{1}^{2}p\left(\epsilon_{2}-\epsilon_{1}\right)\\ \; & =\frac{p\left(\epsilon_{2}-\epsilon_{1}\right)\left[p\epsilon_{1}^{2}\left\{p{\left[2\right]}_{p,q}+{\left[3\right]}_{p,q}\left[{\left[2\right]}_{p,q}-2\right]\right\}+2p\epsilon_{1}\epsilon_{2}\left[{\left[3\right]}_{p,q}-p{\left[2\right]}_{p,q}\right]+p^{2}\epsilon_{2}^{2}{\left[2\right]}_{p,q}\right]}{{\left[2\right]}_{p,q}{\left[3\right]}_{p,q}}. \end{array}$$

Furthermore, for $\kappa \neq \epsilon_{1}$,

(42) $$\begin{array}{cc} {}_{\epsilon_{1}}D_{p,q}H\left(\kappa \right)={\;}_{\epsilon_{1}}D_{p,q}\kappa^{2}= & \frac{{\left(p\kappa +\left(1-p\right)\epsilon_{1}\right)}^{2}-{\left(q\kappa +\left(1-q\right)\epsilon_{1}\right)}^{2}}{\left(p-q\right)\left(\kappa -\epsilon_{1}\right)}\\ \; & =\frac{{\left[2\right]}_{p,q}\kappa^{2}+2\epsilon_{1}\kappa \left\{1-{\left[2\right]}_{p,q}\right\}+\epsilon_{1}^{2}\left\{{\left[2\right]}_{p,q}-2\right\}}{\left(\kappa -\epsilon_{1}\right)}\\ \; & =\frac{\kappa {\left[2\right]}_{p,q}\left(\kappa -\epsilon_{1}\right)-\epsilon_{1}{\left[2\right]}_{p,q}\left(\kappa -\epsilon_{1}\right)+2\epsilon_{1}\left(\kappa -\epsilon_{1}\right)}{\left(\kappa -\epsilon_{1}\right)}\\ & ={\left[2\right]}_{p,q}\left(\kappa -\epsilon_{1}\right)+2\epsilon_{1}. \end{array}$$

Therefore, using the Corollary 3 (iv) with σ=1 and $\eta \left(\epsilon_{2},\epsilon_{1}\right)=\epsilon_{2}-\epsilon_{1}$, we have

$$\begin{array}{cc} \; & \left|\frac{q\epsilon_{1}^{2}+p\epsilon_{2}^{2}}{{\left[2\right]}_{p,q}}-\frac{p\epsilon_{1}^{2}\left\{p{\left[2\right]}_{p,q}+{\left[3\right]}_{p,q}\left[{\left[2\right]}_{p,q}-2\right]\right\}+2p\epsilon_{1}\epsilon_{2}\left[{\left[3\right]}_{p,q}-p{\left[2\right]}_{p,q}\right]+p^{2}\epsilon_{2}^{2}{\left[2\right]}_{p,q}}{{\left[2\right]}_{p,q}{\left[3\right]}_{p,q}}\right|\\ \; & \leq \frac{\left(\epsilon_{2}-\epsilon_{1}\right)2qp^{2}}{{\left[2\right]}_{p,q}^{3}}\max\left(|{}_{\epsilon_{1}}D_{p,q}H\left(\epsilon_{1}\right)\left|,\right|{}_{\epsilon_{1}}D_{p,q}H\left(\epsilon_{2}\right)|\right), \end{array}$$

we get the desired inequality (41).

If we let $q\to 1^{-}$ and p=1, we obtain

$$\left|A\left(\epsilon_{1}^{2},\epsilon_{2}^{2}\right)-L_{2}^{2}\left(\epsilon_{1},\epsilon_{2}\right)\right|\leq \frac{\epsilon_{2}\left(\epsilon_{2}-\epsilon_{1}\right)}{2}.$$

*□*

**Remark** **13.** *Applying the same idea as in Proposition 1 using Theorems 5–8 and their corresponding corollaries, and taking suitable functions, for example, $H\left(x\right)=x^{k},\;k>1$ and $x>0;H\left(x\right)=\frac{1}{x},\;x>0;H\left(x\right)=e^{x},\;x\in R,$ etc., we can obtain several new interesting inequalities using special means. We omit their proofs and the details are left to the interested reader.*

## 7. Conclusions

This research demonstrates some parameterized post quantum trapezoidal and midpoint integral inequalities in terms of the η-quasiconvex based on a post quantum integral identities with a parameter μ∈[0,1]. By choosing different values of parameter μ, we can extract several sub-results from our main results. Further research will focus on parameterized modifications of the left and right parts of Hermite–Hadamard inequality and other well-known mathematical inequalities via (p,q)-integrals.

## Data Availability

Not applicable.
